# *ACS Central Science*: Embracing Breadth in Scope and Scientific Topical Representation

**DOI:** 10.1021/acscentsci.5c01315

**Published:** 2025-09-24

**Authors:** Sofia Garakyaraghi, Kirk S. Schanze

*A* *CS Central Science* was launched by ACS Publications in March of 2015 under the leadership of Editor-in-Chief Carolyn Bertozzi (see the Editorial in the inaugural issue of the journal: 10.1021/acscentsci.5b00090). The journal is envisioned to serve as the vanguard journal for the ACS Publications portfolio, with a unique role as the only “Diamond” open access journal—entirely free to read and free to publish, with no charges for the author or reader. A key aspect of *ACS Central Science* is its scope—it encompasses the broad range of topics that are covered by the ACS Publications journal portfolio. As stated in the scope statement, the journal aims to publish high quality and impactful articles that span the topics relevant to the chemical sciences and related fields. The scope of the journal as stated in the Instructions to Authors (https://pubs.acs.org/page/acscii/about.html) is the following:

...a unique role as the only “Diamond” open access journal—entirely free to read and free to publish, with no charges for the author or reader.

*ACS Central Science* is a multi- and interdisciplinary journal that publishes articles of exceptional quality and interest to the broad chemistry and scientific community. The journal addresses important advances in fundamental areas of chemistry, as well as applied and interdisciplinary research, highlighting the seminal role of chemistry in a wide range of other scientific disciplines. The journal considers submissions in core fields such as, but not limited to

- Analytical, physical, inorganic, and organic chemistry
- Biological and medicinal chemistry, and biotechnology
- Sustainable and environmental chemistry
- Computational and theoretical chemistry
- Materials and nanoscience
- Energy and catalysis
- Chemical engineering
- Earth, atmospheric, and space chemistry
- Chemical education

The journal also publishes inter- and multidisciplinary research applicable to allied fields across the physical sciences, life sciences, engineering, medicine, and beyond.

Given the breadth of the scope statement, one would expect that *ACS Central Science* publishes articles that cover a range of topics that is balanced and represents the broad range of topics described in the scope statement. Yet, when we take a closer look at the Issues and Volumes over the first decade of the journal, a different story emerges. Over these years, *ACS Central Science* has developed a strong presence in areas at the interface of chemistry and biology (e.g., Biochemistry, Chemical Biology, Medicinal Chemistry, etc.). These contributions have been trailblazing reports that have broad and far-reaching impacts reflecting the central role chemistry holds in the medical sciences. This was especially felt during the pandemic era, when several landmark COVID related papers were published in *ACS Central Science* (https://pubs.acs.org/pb-assets/acs-central-science-examples-of-the-exceptional-2021/index.html).^1–10^

However, this strong focus on chemistry and biology has led to an imbalance in topical coverage in *ACS Central Science*. To better understand this trend, we conducted an analysis using Chemical Abstracts (CA) section classifications, comparing *ACS Central Science* with five other leading chemistry journals, including the *Journal of the American Chemical Society.* The results, shown in Figure 1, show that >60% of articles in *ACS Central Science* fall into the broad CA section “Biochemistry.”[Fn fn1] In contrast, the other leading chemistry journals, in aggregate, exhibit a more balanced representation of topics across the traditional areas of the chemical sciences. This represents an opportunity for *ACS Central Science* to more fully realize its founding vision by encouraging submissions from a broader breadth of topics across chemistry and allied fields as the journal continues to grow and evolve.

**Figure 1:**
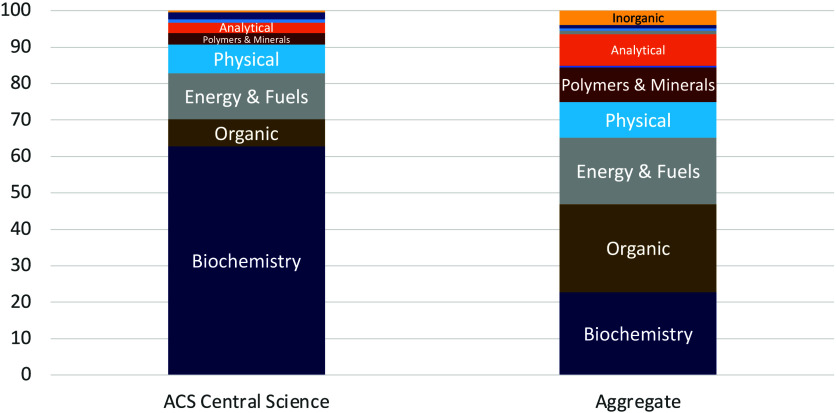
Topical analysis of all papers published in 2024. Left: *ACS Central Science*. Right: Aggregated average of five leading chemistry journals, including *J. Am. Chem. Soc.* Data from Chemical Abstracts.

While several factors may contribute to the current topical distribution in *ACS Central Science*, we suggest that the strong presence of “Biochemistry” articles is largely a result of the journal’s current and past visibility in this area. Because the journal has already published a substantial number of articles in “Biochemistry,” it is perceived by the community as being particularly welcoming to submissions in this field. In turn, this encourages more authors from the “Biochemistry” community to submit their articles. However, this trend is not by design. The editorial team of *ACS Central Science* is committed to expanding the journal’s topical reach and strongly encourages submissions across the full spectrum of the chemical sciences and related fields.

The editorial team of *ACS Central Science* is committed to expanding the journal’s topical reach and strongly encourages submissions across the full spectrum of the chemical sciences and related fields.

As *ACS Central Science* celebrates its 10th anniversary (see editorial: https://pubs.acs.org/doi/10.1021/acscentsci.3c01604), the journal is embracing this milestone as an opportunity to broaden the scope of topics it publishes. Building on a decade of publishing cutting-edge research across the chemical sciences, the journal now seeks to highlight a broader range of contributions that intersect with chemistry. By expanding its focus, *ACS Central Science* aims to reflect the evolving landscape of chemical innovation and to serve as a vibrant platform that shapes the future of chemical sciences.

To steer the journal toward broader topical representation, we must take proactive and strategic steps to engage with the wider scientific community. First, the editors and editorial advisory board should actively “get the word out” that *ACS Central Science* enthusiastically welcomes submissions from all areas of the chemical sciences—especially those underrepresented in the journal’s current portfolio, such as physical chemistry, materials science, catalysis, and chemical engineering. Clear communication through conferences, webinars, social media, and direct outreach can help shift perceptions of the journal’s scope. Second, we can identify and invite leading researchers in these fields to contribute “invited content,” including perspectives, reviews, and research articles, which will both signal our commitment to topical diversity and help seed new areas of growth. These efforts will reinforce *ACS Central Science*’s role as a truly topically balanced and interdisciplinary vanguard journal for ACS Publications.

Following is a nonexhaustive list of areas where the journal would especially welcome an increase in submissions and publications: 1) Inorganic Chemistry and related fields (e.g., catalysis, coordination chemistry, coordination polymers and metal–organic frameworks, inorganic photochemistry, bioinorganic chemistry); 2) Physical Chemistry and related fields (theoretical chemistry and modeling, ultrafast science, physical chemistry of energy science, spectroscopy, single molecule and surface science); 3) Analytical Chemistry and related fields (bioanalytical chemistry, spectroscopy, imaging, environmental analysis); 4) Organic Chemistry (beyond medicinal chemistry, organic catalysis, total synthesis, physical organic chemistry, photochemistry, supramolecular chemistry); 5) Materials Chemistry and Science (fundamental materials discovery, inorganic and organic materials chemistry, applied materials chemistry).

*ACS Central Science* aims to reflect the evolving landscape of chemical innovation and to serve as a vibrant platform that shapes the future of chemical sciences.

We hope this editorial will stimulate authors who have not published in *ACS Central Science* to consider submitting their best works for consideration at the journal. In view of this, the following are some points that authors should consider before submitting a paper. First, because of our mandate to publish a limited number of papers in each issue, authors should be selective about which papers they submit. Authors should review the guidelines (https://researcher-resources.acs.org/publish/author_guidelines?coden=acscii) and keep in mind that the goal of the journal is to publish high impact works that will be broadly interesting to the scientific community and potentially beyond. Second, authors should always include a short cover letter with their submission that includes a statement that clearly describes: 1) the *novelty and significance* of the work that is reported; 2) why the paper is suitable for the scope of *ACS Central Science*. Authors should be aware that a two- to three-page cover letter that merely repeats content from the abstract, introduction, or conclusion is not useful to the editors.

We look forward to seeing *ACS Central Science* continue to grow in impact and evolve to reflect the full richness of the broad chemistry community and are excited about the journal’s role to shape the future of chemistry.

## Footnotes

a Here “Biochemistry” refers to the CA section analysis topic which includes 20 different subtopics. See: https://www.cas.org/training/lesson/sections-ca for details.
